# Addressing the Binding Mechanism of the Meprin and TRAF-C Homology Domain of the Speckle-Type POZ Protein Using Protein Engineering

**DOI:** 10.3390/ijms242417364

**Published:** 2023-12-11

**Authors:** Awa Diop, Paola Pietrangeli, Valeria Pennacchietti, Livia Pagano, Angelo Toto, Mariana Di Felice, Sara Di Matteo, Lucia Marcocci, Francesca Malagrinò, Stefano Gianni

**Affiliations:** 1 Laboratory Affiliated to Istituto Pasteur Italia—Fondazione Cenci Bolognetti, Dipartimento di Scienze Biochimiche “A. Rossi Fanelli”, Sapienza Università di Roma, Piazzale Aldo Moro 5, 00185 Rome, Italy; awa.diop@uniroma1.it (A.D.); paola.pietrangeli@uniroma1.it (P.P.); sara.dimatteo@outlook.it (S.D.M.); lucia.marcocci@uniroma1.it (L.M.); 2 Department of Life, Health and Environmental Sciences, University of L’Aquila, 67100 Coppito, Italy

**Keywords:** kinetics, protein–protein interactions, MATH domain, speckle-type POZ protein, site-directed mutagenesis

## Abstract

Protein–protein interactions play crucial roles in a wide range of biological processes, including metabolic pathways, cell cycle progression, signal transduction, and the proteasomal system. For PPIs to fulfill their biological functions, they require the specific recognition of a multitude of interacting partners. In many cases, however, protein–protein interaction domains are capable of binding different partners in the intracellular environment, but they require precise regulation of the binding events in order to exert their function properly and avoid misregulation of important molecular pathways. In this work, we focused on the MATH domain of the E3 Ligase adaptor protein SPOP in order to decipher the molecular features underlying its interaction with two different peptides that mimic its physiological partners: Puc and MacroH2A. By employing stopped-flow kinetic binding experiments, together with extensive site-directed mutagenesis, we addressed the roles of specific residues, some of which, although far from the binding site, govern these transient interactions. Our findings are compatible with a scenario in which the binding of the MATH domain with its substrate is characterized by a fine energetic network that regulates its interactions with different ligands. Results are briefly discussed in the context of previously existing work regarding the MATH domain.

## 1. Introduction

Protein–protein interaction (PPI) domains play crucial roles in diverse biological processes through the recognition of defined consensus motifs. PPIs are involved in the ubiquitin proteasome system (UPS), which is the central mechanism used by eucaryotic cells to maintain protein homeostasis [1,2]. Protein ubiquitination mediates such a system, as ubiquitin binds covalently to the substrate via the action of trienzyme cascades (E1, E2, and E3) [3]. E3 ubiquitin ligases intervene in the last step by ligating the lysine of the target substrate with the C-terminal glycine of ubiquitin to form an isopeptide bond [4,5]. There are over 600 E3 ligases identified in human cells [4,6], and they are divided into four families (HECT-type, U-box-type, RING-finger-type, RBR-type) [7]. E3 ligases, due to their involvement in the regulation of diverse biological and cellular processes, are considered as multifunctional enzymes; thus, they are promising therapeutic targets for anticancer drug development [8,9,10].

**Figure 1:**
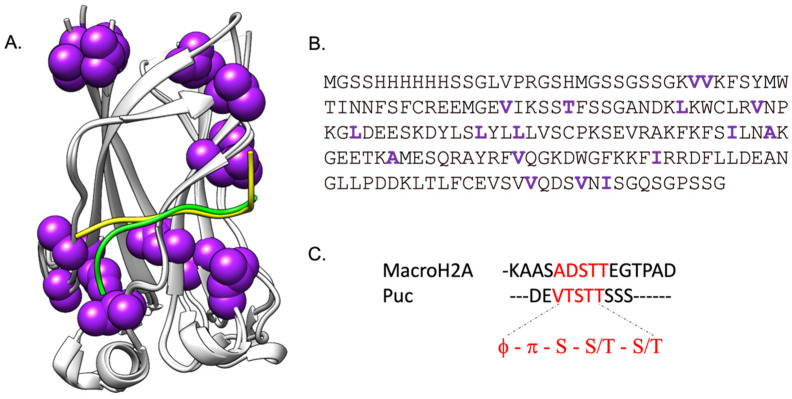
Mapping of the allosteric network of the SPOP MATH domain in complex with the peptides Puc (green) and MacroH2A (yellow). The amino acid residues in SPOP MATH that were subjected to mutagenesis in this work are highlighted in purple in the structure (Panel (**A**)) and sequence (Panel (**B**)). This representation is made using UCSF Chimera, with the PDB structure 3HQL (Puc) and 3HQH (MacroH2A). Panel (**C**) represents the alignment of the peptide sequences that highlight the consensus-conserved motifs (φ-π-S-S/T-S/T) in red. The protein–peptide contacts found in the pdb have been also compared using the program PISA (https://www.ebi.ac.uk/pdbe/pisa/) (accessed on 25 November 2023), which confirmed that the two peptides interact with SPOP MATH in a similar manner involving residues 71, 77, 88, 103, 118–121, 124, and 129–136 in the case of Puc and residues 71, 73, 77, 88, 103, 118.121, 124, and 129–135 in the case of MacroH2A.

Cullin-3 (Cul-3) is the most common E3 ligase involved in substrate degradation [11]. For instance, Cul-3 interacts with the BTB (Broad-Complex, Tramtrack, and Bric à brac) domain of SPOP (Speckle-type POZ protein), while its MATH (Meprin and TRAF-C Homology) domain recruits the substrates for ubiquitination [12]. SPOP protein is an E3 ubiquitin ligase adaptor protein that is involved in various cellular processes, including protein ubiquitination, degradation, and apoptosis. SPOP recruits its substrates through its MATH domain, which recognizes the substrates through a short linear motif called the SBC (SPOP Binding Consensus) motif [13]. The SBC motif consists of a non-polar residue, followed by a polar residue, a serine, and then a serine or threonine (Figure 1) [13]. Furthermore, SPOP is implicated in several malignancies, being mutated in different types of cancers [14]. Indeed, mutations of this protein in its MATH domain are major causes of prostate and endometrial cancers, whereas an overexpression is observed in the case of kidney cancer [14].

The interaction of the MATH domain with different substrates, such as the phosphatase Puc [13], the chromatin component MacroH2A [13,15], and the dual specificity phosphatase, PTEN [16], has been investigated from structural and kinetic perspectives, allowing the identification of the residues involved in the binding process [17]. Importantly, the presence of a conserved binding pocket has been observed both by crystallography and NMR. However, there is a lack of information regarding the mechanism underlying the ability of the MATH domain to accommodate different ligands in its binding pocket. This property suggests the presence of a fine regulation of the binding events, which might not be pinpointed by a purely structural characterization.

In this work, by employing stopped-flow kinetic binding experiments in combination with extensive site-directed mutagenesis (Figure 1), we compared the effect of mutations of the SPOP MATH domain on the binding mechanism to its physiological binders Puc and MacroH2A (Figure 1C). Our data highlighted the contribution of different residues, some of which were located far from the binding pocket, which may be part of a sparse energetic network that finely regulates the binding capabilities of the MATH domain. Results are discussed in the light of previous studies performed on the MATH domain.

## 2. Results and Discussion

### 2.1. Long Range Interactions between SPOP MATH and Its Physiological Binders Govern the Binding Process

A common method used to infer the features underlying molecular interactions is based on the perturbation of a protein system through extensive site-directed mutagenesis and monitoring of the effect of such perturbation on the kinetics of the binding reaction. Thus, we generated and purified 40 site-directed mutants of the SPOP MATH domain and carried out time-resolved kinetic binding experiments. Unfortunately, of the 40 site-directed mutants generated, a large fraction did not express a high yield and, consequently, we were able to record reliable binding traces with only 17 site-directed MATH mutant variants. Conservative mutations have been designed by following the phi-value analysis rules [18]. As described in our previous work [17], we investigated the binding process between monomeric SPOP MATH domain variants and the peptides mimicking its substrates Puc and MacroH2A, which were grafted from a dansyl group at their N-termini, by taking advantage of the three tryptophan residues of the MATH domain in order to spectroscopically monitor the binding events through a FRET (Forster Resonance Energy Transfer) signal [17]. A sketch summarizing the experimental setup is reported in Figure S2. We could not measure the binding with PTEN owing to its lower affinity with the latter peptide.

When investigating the effect of site-directed mutants on the binding mechanism of the SPOP MATH domain to its substrates, it is important to verify that all the variants tested maintain a native-like structure. To confirm that, we performed equilibrium denaturation experiments using urea as a chaotropic agent. For all 17 site-directed mutants tested, we observed that all variants are native in the absence of denaturant and display a similar stability when compared to the wildtype protein (Figure S1 and Table S1 in the Supplementary Data File).

**Figure 2:**
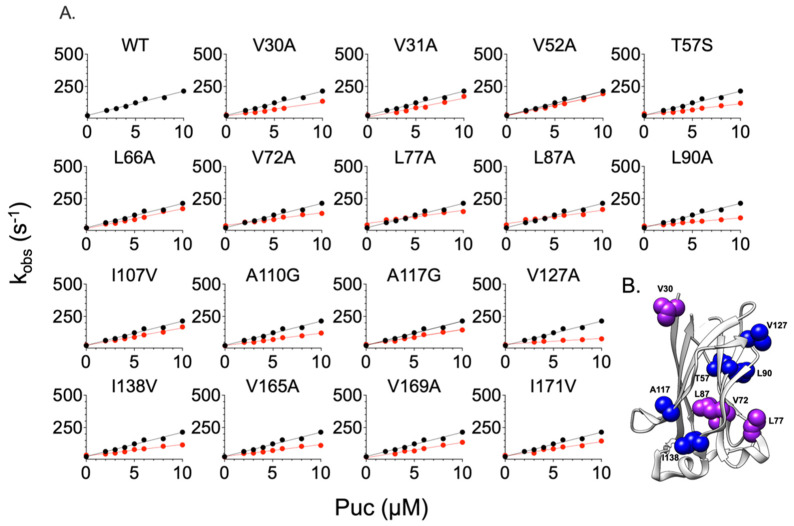
Kinetic binding experiments between SPOP MATH variants and the peptide mimicking Puc. Panel (**A**) represents the binding of MATH mutant variants with Puc. Each line represents the best fit to a linear equation. In all plots, the black and red dots represent the wt and mutant variants, respectively. The related kinetic parameters are listed in Table 1. The representation in panel (**B**) is made using UCSF Chimera version 1.16 and PDB code 3HQL. The residues significantly affecting the kinetics parameters k_on_ are displayed in blue, whereas the residues affecting both k_on_ and k_off_ are highlighted in purple. The residues V169 and I171 are not present in the pdb structure.

**Figure 3:**
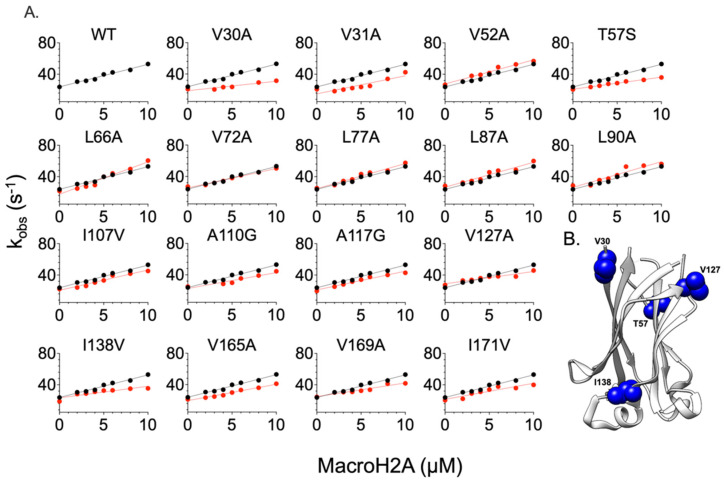
Kinetic binding experiments between SPOP MATH variants and the peptide mimicking MacroH2A. Panel (**A**) represents the binding between a MATH mutant variant and MacroH2A. The lines represent the best fit with a linear equation. In all plots, the black and red dots represent the wt and mutant variants, respectively. The related kinetic parameters are listed in Table 2. Panel (**B**) represents the allosteric network affecting the binding to MacroH2A. The residues inducing a change in the activation and equilibrium free energy of the binding are highlighted in blue. This representation is made using UCSF Chimera version 1.16 and PDB code 3HQH. The residues V169 and I171 are not present in the pdb structure.

We monitored the kinetic binding events via a time-resolved stopped-flow apparatus by rapidly mixing a fixed concentration of MATH domain variants (1 µM) with increasing concentrations of dansylated peptides (Puc or MacroH2A, from 2 µM to 10 µM). The buffers used were 50 mM HEPES, at physiological pH of 7.2, and the binding occurred at 298 K. All the binding traces obtained were satisfactorily fitted with a single exponential equation (see Materials and Methods section). A typical fluorescence time course may be observed in Figure S3. The fitting allows us to calculate the binding observed rate constants (k_obs_) that are plotted versus different concentrations of dansylated peptides for each SPOP MATH mutant variant, and compare them with SPOP MATH wt. The obtained data were fitted with a linear equation, with the microscopic association (k_on_) and dissociation (k_off_) rate constants represented by the slope and *y*-axis intercept, respectively. Because of the higher experimental error related to the extrapolation of the k_off_, we proceeded to a displacement experiment for each binding experiment in order to determine the real k_off_ and then calculate the dissociation constant, which was the ratio between the k_off_ and k_on_. The displacement experiment consists of rapidly mixing (1 µM) a SPOP MATH variant in a complex with a fixed concentration of dansylated peptide (8 µM), versus a higher excess of non-dansylated peptide (50 µM) [19]. The plots corresponding to the k_obs_ over the concentration of dansylated peptides are summarized in Figure 2 and Figure 3 for Puc and MacroH2A, respectively. In addition, the thermodynamic parameters associated to each plot are reported in Table 1 and Table 2 for Puc and MacroH2A, respectively.

In the case of the binding of SPOP MATH variants to dansylated Puc (Figure 2 and Table 1), the substitutions V31A, V52A, L66A, and I107V do not induce any significant change in the microscopic association and dissociation rate constants, compared to the wildtype (Figure 2, Table 1). However, we observed a decrease in the microscopic association rate constant of a factor of at least 1.5, for V30A, T57S, V72, L77, L87, L90A, A110G, A117G, V127A, I138V, V165A, V169A, and I171V (Figure 2, Table 1), suggesting that these residues may be involved in long-range interactions that lead to the formation of the complex MATH: Puc. A closer look into the incidence of the mutations on the microscopic dissociation rate constants shows that the substitutions V72A, L77A, and L87A increase the k_off_ by a factor of at least 1.5 (Figure 2, Table 1). These findings suggest that the residues V72, L77, and L87 are potentially involved in the early and late binding events, even though they are located far from the binding pocket (Figure 2, Table 1). Concerning the dissociation constant, all SPOP MATH mutant variants have lower affinity to Puc compared to the wildtype. Of interest, it is important to note that none of the probed mutations induced a detectable stabilization of the complex, indicating that the protein appears to be optimized to bind its specific ligand, even when challenged with substitutions that are located far from the binding site.

In the case of the binding of SPOP MATH variants with dansylated MacroH2A, we realized that the SPOP MATH variants V31A, V52A, L66A, V72A, L77A, L87A, L90A, I107V, A110G, A117G, V127A, V165A, V169A, and I171V reflect unsignificant variations on k_on_ and k_off_ values, compared to the wildtype. Indeed, the measured k_off_ corresponding to each mutant remains essentially unchanged when compared to the wildtype domain (Figure 3, Table 2). Importantly, the V30A, V127A, and I138V substitutions significantly affect the early events of recognition of the ligand, with a k_on_ of at least 1.5 times lower than the wildtype (Figure 3, Table 2). The analysis of change in activation free energy, between the wildtype and each mutant variant, shows that the substitutions V31A, V52A, L66A, V72A, L77A, L87A, L90A, I107V, A110G, A117G, V165A, V169A, and I171A do not significantly affect the stabilization of the complex with MacroH2A, as ∆∆G_eq_ < |0.35| kcal mol^−1^.

To sum up, we can conclude that whilst the residues V72, L77, L87, L90, and A117 show a significant effect when SPOP MATH is analyzed in its binding to Puc, the residues V30, T57, V127, and I138 contribute to both complexes. In addition, the microscopic dissociation rate constant of the binding of MATH mutants to Puc appears more perturbed than in the binding to MacroH2A, suggesting the selective binding of SPOP MATH to each of the two substrates is inherently linked to the microscopic association rate constant, thus to the early recognition of the substrates. As described in the literature, the binding domain of MATH interacts with a multitude of binding partners; however, little is known about the selective interaction of one to another [13,16].

### 2.2. Comparing the Effect of Site-Directed Mutagenesis on the Binding of Two Different Substrates

The MATH domain is a multifunctional protein that interacts with different substrates that are involved in various biological processes. The results described above allow the identification of energetic networks that are stabilizing the complex of SPOP MATH with either Puc or MacroH2A. In both cases, the structural distribution of the networks appears sparse and unevenly distributed within the protein domain. It should be noticed that whilst the three-dimensional structure of MATH appears essentially superposable when solved in a complex with the two different substrates, a purely structural approach might not allow us to fully address the underlying molecular mechanism of its binding to the substrates.

**Figure 4:**
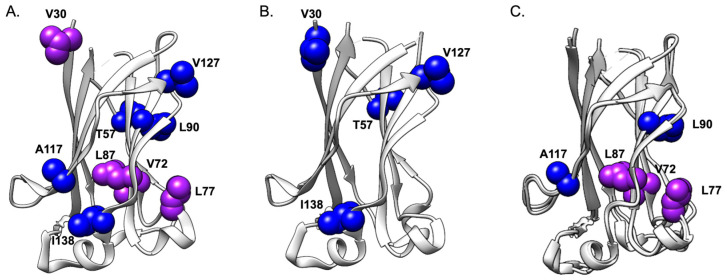
Mapping allosteric networks affecting Puc and MacroH2A interactions to MATH. The panels (**A**,**B**) represent the allosteric network affecting the binding parameters k_on_ (blue) k_off_ (red) or both (purple) of SPOP MATH to Puc and MacroH2A, respectively. Panel (**C**). represents the energetic network of SPOP MATH selective binding to Puc over MacroH2A. The MATH variants corresponding to the ratio of ∆∆G_eq_ ^Puc^/∆∆G_eq_ ^MacroH2A^ > 2 (Table 3) are pinpointed in blue and purple due to their impact on the k_on_, or both k_on_ and k_off_ kinetic parameters, respectively. Panel (**C**) results from the superimposition of the PDB structures 3HQL (Puc) and 3HQH (MacroH2A). These representations are made using UCSF Chimera software, version 1.16. The residues V169 and I171 are not present in the pdb structure.

In order to investigate the selectivity of SPOP MATH binding to Puc over MacroH2A, we calculated the ratio of the change in equilibrium free energy between the wildtype and each mutant variant, binding to Puc over MacroH2A (Table 3 and Figure 4C). To minimize errors, we considered only the mutants inducing a change in the stability of the complex superior or equal to 0.35 kcal mol^−1^. The analysis of the ratio ∆∆G_eq_^Puc^/∆∆G_eq_^MacroH2A^ is summarized in Table 3 and Figure 4. Inspection of the obtained values indicate that the residues V72, L77, L87, L90, A117, and I171 appear to be optimized for Puc rather than MacroH2A, displaying a value of ∆∆G_eq_^Puc^/∆∆G_eq_^MacroH2A^ higher than 2. On the basis of these observations, it is tempting to speculate that these residues are critical for fine-tuning the binding selectivity of the aforementioned domain.

### 2.3. Properties of the Binding Transition States

**Figure 5:**
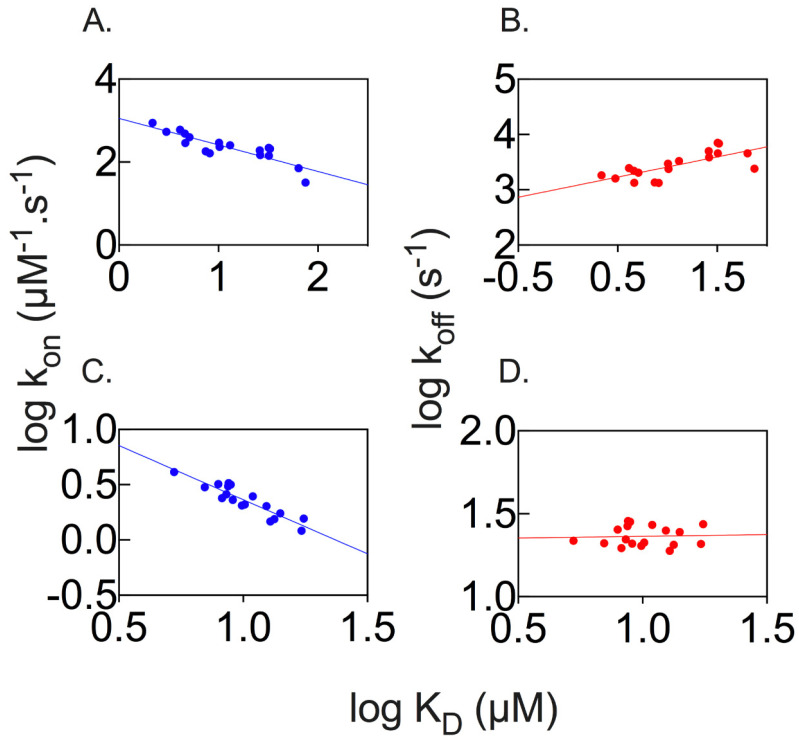
Linear free energy relationship for the binding of MATH wildtype and mutant variants to the peptides Puc (panels (**A**,**B**)) and MacroH2A (panels (**C**,**D**)). Panels (**A**,**C**) represent the dependences of the microscopic association rate constant k_on_ on the equilibrium dissociation constant. Panels (**B**,**D**) represent the dependences of the microscopic dissociation rate constant k_off_ on the equilibrium dissociation constant. The blue and red dots represent the dependences of k_on_ or k_off_ on the equilibrium dissociation constant (K_D_), respectively.

A powerful method used to address the mechanism of binding between ligands is to study the effect of structural perturbation on the transition and ground states. This approach, classically denoted as Leffler or Linear Free Energy Relationship (LFER) analysis, aims at analyzing the correlation of the change in activation free energy to the change in equilibrium free energy; LFER analysis has been used in organic chemistry, protein folding, enzymology, and protein–protein interaction studies to decipher the position of the transition state of a reaction along the reaction coordinates [20,21,22,23,24,25]. The analysis of the dependences of the microscopic association and dissociation rate constants over the equilibrium constant of each MATH variant to Puc and MacroH2A are reported in Figure 5. Interestingly, in both cases, the mutational data yield a linear LFER plot. In analogy to what was previously discussed regarding protein folding, this observation might be taken as a hallmark of cooperativity and suggests that not only the residues located in the binding pocket, but all the probed residues taken, are involved in the binding of the ligand, with the transition state resembling a distorted version of the native state.

LFER analysis of the binding of Puc and MacroH2A returns a linear behavior in both cases. It is important to observe that, in the former, the slope of log k_on_ vs. log K_D_ is equal to −0.64 ± 0.09, and log k_off_ versus log K_D_ is equal to 0.36 ± 0.09; in the case of the latter, log k_on_ vs. log K_D_ is equal to −0.98 ± 0.11 and log k_off_ versus log K_D_ is equal to 0.02 ± 0.11. Hence, while in the case of MacroH2A the effects of the designed mutations appear to affect only the early events of binding, in the case of Puc, they have an effect on both the recognition and stabilization of the complex events, as mirrored by a detectable dependence on both k_on_ and k_off_. These finding suggest that the different contributions measured may be at the root of the selectivity mechanism of MATH for different ligands.

## 3. Materials and Methods

### 3.1. Protein Expression and Purification

A construct encoding SPOP MATH domain (MATH domain where cysteine residues have been substituted for to Serines) was subcloned in a pHTP1 plasmid vector and then transformed in *Escherichia coli* BL21 cells (DE3). Bacterial cells were grown in an LB medium, supplemented with 30 μg/mL of kanamycin, at 37 °C until OD_600_ = 0.7−0.8, and then protein expression was induced with 1 mM IPTG. After induction, cells were grown at 25 °C overnight and then collected by centrifugation. To purify the His-tagged protein, the pellet was resuspended in a buffer made of 50 mM Tris-HCl, 300 mM NaCl, 10 mM Imidazole, and pH 7.5, with the addition of an antiprotease tablet (cOmplete, EDTA-free, Roche Diagnostics GmbH, Mannheim, Germany), then it was sonicated and centrifuged. The soluble fraction taken from bacterial cell lysate was loaded onto a nickel-charged HisTrap Chelating HP (GE Healthcare Bio-Sciences AB, Uppsala, Sweden) column equilibrated with 50 mM Tris-HCl, 300 mM NaCl, 10 mM Imidazole, and pH 7.5. Protein was then eluted with a gradient from 0 to 1 M imidazole by using an ÄKTA-prime system. Fractions containing the protein were collected and the buffer was exchanged to 50 mM Tris-HCl, 300 mM NaCl, and pH 7.5. The purity of the protein was analyzed through SDS-PAGE (Figure S4, Supplementary Data File). Forty site-directed mutants of the SPOP MATH domain were generated using an NZYTech mutagenesis kit (NZYTech, Lisbon, Portugal), according to manufacturer instructions, and were expressed and purified according to the protocol described above. The designed site-directed mutants are conservative as Alanine, Valine, Isoleucine, Leucine, and Threonine are substituted to Glycine, Alanine, Valine, Alanine, and Serine, respectively, and they follow the phi-value analysis rule described by Sato and Fersht in 2004. Peptides mimicking SPOP MATH substrates, Puc (sequence DEVTSTTSSS), and MacroH2A (sequence KAASADSTTEGTPAD), with and without the dansyl N-terminal modification, were purchased from GenScript (GenScript Biotech, Rijswijk, The Netherlands).

### 3.2. Stopped-Flow Binding Experiments

Kinetic binding experiments were performed on an Applied Photophysics DX-17MV stopped-flow apparatus (Applied Photophysics, Leatherhead, UK). Pseudo-first order binding experiments were performed by mixing a constant concentration (1 μM) of MATH domain with increasing Dansylated peptide concentrations, from 2 to 10 μM of Puc and MacroH2A. Samples were excited at 280 nm, and the emission fluorescence was recorded by using a bandpass 320 nm cutoff filter. Experiments were performed at 298 K. For each acquisition, 3 to 5 traces were collected, averaged, and satisfactorily fitted to a single exponential equation.

k_obs_ = k_on_ [Peptide] + k_off_

### 3.3. Stopped-Flow Displacement Experiments

Microscopic dissociation rate constants (*k*_off_) were directly measured by performing displacement experiments on an Applied Photophysics DX-17MV stopped-flow apparatus (Applied Photophysics, Leatherhead, UK). A preincubated complex of MATH domain and dansylated peptides at a constant concentration (both 8 µM) was rapidly mixed with an excess of non-dansylated peptides (50 μM). Samples were excited at 280 nm and fluorescence emission was collected by using a 330 ± 30 nm bandpass filter. Experiments were performed at 298 K in the same buffer used for binding experiments. The observed rate constants were calculated based on the average of five single traces. Observed kinetics were consistent with a single exponential decay.

### 3.4. Equilibrium Denaturation Experiments

Equilibrium unfolding experiments were performed on a Fluoromax single photon counting spectrofluorometer (Jobin-Yvon, Newark, NJ, USA). Each MATH domain variant was excited at 280 nm and emission spectra were recorded between 300 and 400 nm, at increasing urea concentrations. Experiments were performed with the protein at a constant concentration of 1 µM at 298 K, using a quartz cuvette with a path length of 1 cm. The buffer used for all experiments contains 50 mM HEPES at pH 7.2.

$$Y_{obs}=\frac{(Y_{N}+\alpha_{N}\left[Urea\right])+(Y_{D}+\alpha_{D}\left[Ureal\right])\;e^{\frac{m_{D-N}\left(\left[Urea\right]-{\left[Urea\right]}_{1/2}\right)}{RT}}}{1+e^{\frac{m_{D-N}\left(\left[Urea\right]-{\left[Urea\right]}_{1/2}\right)}{RT}}}$$

where: Y_obs_ is the observed fluorescence signal; Y_N_ and Y_D_ are the fluorescence signals of the native and denaturated states, respectively; $\alpha_{N}=\frac{\partial \;Y_{N}}{\partial \;\left[Urea\right]}$ and $\alpha_{D}=\frac{\partial \;Y_{D}}{\partial \;\left[Urea\right]}$ **;** [Urea]_1/2_ is the denaturant concentration at which the protein is 50% unfolded.

## 4. Conclusions

The specific interactions between protein–protein recognition domains and their physiological partners are critical for the metabolism of cells. In this context, it is of special importance to study how versatile protein domains, such as SPOP MATH, may achieve specificity for different targets while displaying a simple globular structure. The results highlighted above exemplify how, in the case of a MATH domain, binding affinity is modulated by sparse energetic networks that are distributed unevenly within the domain architecture. This finding is of particular importance, given that the SPOP MATH shows negligible conformational changes upon binding, and residues that are critical to stabilize the complex cannot be necessarily deduced from structural studies only. Notably, these kinds of sparse energetic networks have been previously observed on other protein–protein interaction domains, such as SH2 [26], and PDZ [27] domains, indicating that they may likely represent a general property of this class of proteins. Moreover, the analysis of the LFER plots reveal that, whilst both ligands conform to a linear dependence, there is a detectable change in the contribution of mutations to k_on_ and k_off_ to the stability of the complex with the two different peptides. This finding suggests that there is a substantial change in residues involved in the early and late events of binding when SPOP MATH recognizes either Puc or MacroH2A, and the transition states of the two binding reactions are characterized by a different degree of similarity with respect to the bound state. We suggest that the conformational plasticity highlighted in this study, mediated by long-range interactions, represents an additional mechanism for regulation of the MATH domain, and it is capable of fine-tuning its binding capability. Future work on other MATH domains will provide additional information about the generality of such precise regulation.

## Figures and Tables

**Table 1:** Kinetic binding parameters for SPOP MATH mutant variants in their interaction with dansylated Puc. The changes in free energy variations are calculated according to the following equations: ∆∆G_#_ = −RT ln (k_on_^wt^/k_on_^mut^); ∆∆G_eq_ = −RT ln (K_D_^wt^/K_D_^mut^).

	Puc				
MATH Variants	k_on_ (µM^−1^ s^−1^)	k_off_ (s^−1^)	K_D_ (µM)	∆∆G_#_ (kcal mol^−1^)	∆∆G_eq_ (kcal mol^−1^)
WT	19 ± 1	26.1 ± 0.1	1.4 ± 0.4	-	-
V30A	10.66 ± 0.85	29.29 ± 0.55	2.74 ± 0.1	0.34 ± 0.08	0.40 ± 0.04
V31A	14.64 ± 1.5	28.36 ± 0.35	1.94 ± 0.1	0.15 ± 0.10	0.19 ± 0.06
V52A	16.10 ± 0.68	29.71 ± 0.40	1.85 ± 0.05	0.10 ± 0.04	0.16 ± 0.03
T57S	8.60 ± 0.66	38.8 ± 0.5	4.51 ± 0.09	0.47 ± 0.08	0.69 ± 0.02
L66A	15.31 ± 0.78	24.63 ± 0.1	1.61 ± 0.05	0.13 ± 0.05	0.08 ± 0.03
V72A	9.80 ± 0.56	40.35 ± 0.15	4.12 ± 0.06	0.39 ± 0.06	0.64 ± 0.01
L77A	10.16 ± 0.65	46.34 ± 0.50	4.56 ± 0.09	0.37 ± 0.08	0.70 ± 0.02
L87A	10.41 ± 1	46.90 ± 0.15	4.51 ± 0.12	0.35 ± 0.12	0.69 ± 0.02
L90A	6.38 ± 0.43	38.78 ± 0.25	6.09 ± 0.06	0.64 ± 0.07	0.87 ± 0.01
I107V	13.46 ± 0.74	27.33 ± 0.35	2.03 ± 0.05	0.20 ± 0.05	0.22 ± 0.03
A110G	9.58 ± 0.50	22.90 ± 0.20	2.39 ± 0.06	0.40 ± 0.05	0.32 ± 0.03
A117G	11.77 ± 0.60	32.16 ± 0.30	2.73 ± 0.06	0.28 ± 0.05	0.39 ± 0.02
V127A	4.51 ± 0.30	29.39 ± 0.50	6.52 ± 0.09	0.85 ± 0.09	0.91 ± 0.01
I138V	8.74 ± 0.55	36.12 ± 0.40	4.13 ± 0.08	0.46 ± 0.06	0.64 ± 0.02
V165A	9.14 ± 0.68	22.74 ± 1	2.49 ± 0.08	0.43 ± 0.08	0.34 ± 0.05
V169A	11.70 ± 1	22.76 ± 1	1.95 ± 0.10	0.29 ± 0.09	0.19 ± 0.06
I171V	11.06 ± 1	33.74 ± 1	3.05 ± 0.12	0.32 ± 0.09	0.46 ± 0.04

**Table 2:** Kinetic binding parameters for SPOP MATH mutant variants’ interaction with dansylated MacroH2A. The changes in free energy variations are calculated according to the following the equations: ∆∆G_#_ = −RT. ln (k_on_^wt^/k_on_^mut^); ∆∆G_eq_ = −RT.ln (K_D_^wt^/K_D_^mut^).

	MacroH2A				
MATH Variants	k_on_ (µM^−1^ s^−1^)	k_off_ (s^−1^)	K_D_ (µM)	∆∆G_#_ (kcal mol^−1^)	∆∆G_eq_ (kcal mol^−1^)
WT	3 ± 2	21 ± 2.0	7.8 ± 0.4	-	-
V30A	1.21 ± 0.20	20.78 ± 1.5	17.17 ± 0.25	0.54 ± 0.20	0.53 ± 0.01
V31A	2.30 ± 0.30	20.87 ± 3.0	9.07 ± 0.4	0.16 ± 0.13	0.15 ± 0.03
V52A	3.06 ± 0.25	26.61 ± 1.5	8.70 ± 0.15	−0.01 ± 0.08	0.13 ± 0.02
T57S	1.54 ± 0.1	20.54 ± 0.50	13.42 ± 0.09	0.40 ± 0.07	0.38 ± 0.01
L66A	4.12 ± 0.40	21.72 ± 2.0	5.30 ± 0.21	−0.19 ± 0.10	−0.17 ± 0.03
V72A	2.48 ± 0.15	27.12 ± 0.5	10.85 ± 0.07	0.11 ± 0.06	0.26 ± 0.01
L77A	3.20 ± 0.30	25.40 ± 1.5	7.94 ± 0.15	−0.04 ± 0.09	0.07 ± 0.02
L87A	3.17 ± 0.40	28.24 ± 2.0	8.40 ± 0.20	−0.03 ± 0.13	0.14 ± 0.02
L90A	3.27 ± 0.44	28.57 ± 2.5	8.66 ± 0.23	−0.05 ± 0.13	0.13 ± 0.03
I107V	2.59 ± 0.25	22.17 ± 1.5	8.53 ± 0.20	0.09 ± 0.10	0.12 ± 0.02
A110G	2.02 ± 0.30	25.06 ± 2	12.41 ± 0.24	0.23 ± 0.15	0.34 ± 0.02
A117G	2.39 ± 0.20	19.63 ± 1	8.18 ± 0.15	0.13 ± 0.08	0.09 ± 0.02
V127A	1.56 ± 10	27.34 ± 1	17.53 ± 0.20	0.39 ± 0.13	0.54 ± 0.01
I138V	1.47 ± 0.30	18.89 ± 2	12.60 ± 0.30	0.42 ± 0.12	0.36 ± 0.01
V165A	2.09 ± 0.20	21.21 ± 1	11.46 ± 0.15	0.21 ± 0.08	0.22 ± 0.01
V169A	1.74 ± 0.20	24.53 ± 1.5	11.60 ± 0.10	0.32 ± 0.06	0.41 ± 0.01
I171V	2.05 ± 0.35	20.26 ± 3	9.18 ± 0.30	0.22 ± 0.05	0.20 ± 0.01

**Table 3:** Ratio of variation of free energy associated with the equilibrium dissociation constant between the wildtype and mutant variants of SPOP MATH, binding to Puc versus MacroH2A.

MATH Variants	∆∆G_eq_^Puc^ (kcal mol^−1^)	∆∆G_eq_^MacroH2A^ (kcal mol^−1^)	∆∆G_eq_^Puc^/∆∆G_eq_^MacroH2A^
WT	-	-	-
V30A	0.40 ± 0.04	0.53 ± 0.01	0.75 ± 0.03
T57S	0.69 ± 0.02	0.38 ± 0.01	1.82 ± 2.12
V72A	0.64 ± 0.01	0.26 ± 0.01	2.42 ± 0.01
L77A	0.70 ± 0.02	0.07 ± 0.02	9.40 ± 0.01
L87A	0.69 ± 0.02	0.14 ± 0.02	4.85 ± 0.01
L90A	0.87 ± 0.01	0.13 ± 0.03	6.62 ± 0.05
A117G	0.39 ± 0.02	0.09 ± 0.02	4.18 ± 0.02
V127A	0.91 ± 0.01	0.54 ± 0.01	1.68 ± 0.01
I138V	0.64 ± 0.02	0.36 ± 0.01	1.78 ± 0.01
I171V	0.46 ± 0.04	0.20 ± 0.01	2.26 ± 0.02

## Data Availability

Data is contained within the article and supplementary materials.

## Supplementary Materials

The following supporting information can be downloaded at: https://www.mdpi.com/article/10.3390/ijms242417364/s1.
