# Supplementary material for: Addressing the Binding Mechanism of the Meprin and TRAF-C Homology Domain of the Speckle-Type POZ Protein Using Protein Engineering
Source: Int J Mol Sci. 2023 Dec 11;24(24):17364. doi: 10.3390/ijms242417364 (PMC10743451; doi:10.3390/ijms242417364)
Supplement: Supplementary file 1 [file ijms-24-17364-s001.zip › ijms-2720383-supplementary.pdf]

Supplementary data

Addressing the Binding Mechanism of the Meprin and TRAF-C Homology Domain of the Speckle-Type POZ Protein Using Protein Engineering

Awa Diop [1], Paola Pietrangeli [1], Valeria Pennacchietti [1], Livia Pagano [1], Angelo Toto [1], Mariana Di Felice [1], Sara Di Matteo [1], Lucia Marcocci [1], Francesca Malagrinò [2] and Stefano Gianni [1,*]

[1] Laboratory Affiliated to Istituto Pasteur Italia—Fondazione Cenci Bolognetti, Dipartimento di Scienze Biochimiche "A. Rossi Fanelli", Sapienza Università di Roma, Piazzale Aldo Moro 5, 00185 Rome, Italy; awa.diop@uniroma1.it (A.D.); paola.pietrangeli@uniroma1.it (P.P.); valeria.pennacchietti@uniroma1.it (V.P.); livia.pagano@uniroma1.it (L.P.); angelo.toto@uniroma1.it (A.T.); mariana.difelice@uniroma1.it (M.D.F.); sara.dimatteo@outlook.it (S.D.M.); lucia.marcocci@uniroma1.it (L.M.)

[2] Department of Life, Health and Environmental Sciences, University of L'Aquila, 67100 Coppito, Italy; francesca.malagrino@univaq.it

* Correspondence: stefano.gianni@uniroma1

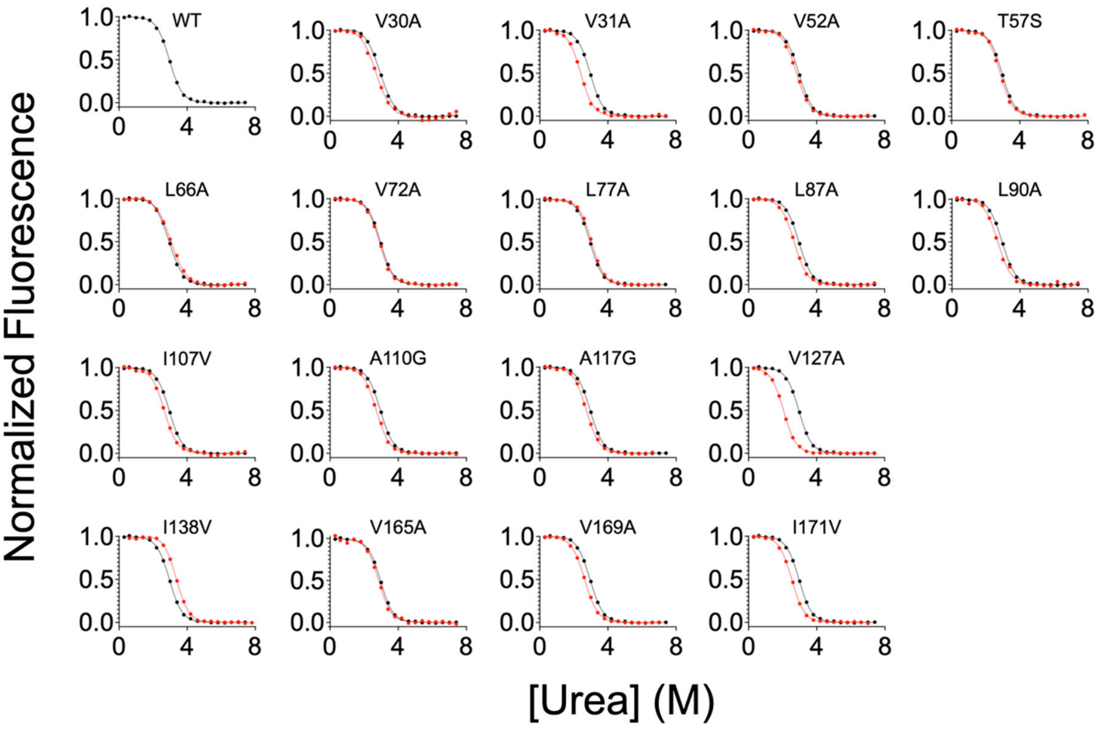

Figure S1. Equilibrium Unfolding experiments of SPOP MATH mutant variants. In black and red are represented the Equilibrium Unfolding of SPOP MATH domain

and its 17 site-directed mutant variants, respectively. All the experiments are carried out at 298 K, with 50 mM HEPES, pH 7.2, using a Fluoromax single photon counting spectrofluorometer. The solid lines are the best fit to an equation describing a two state unfolding mechanism.

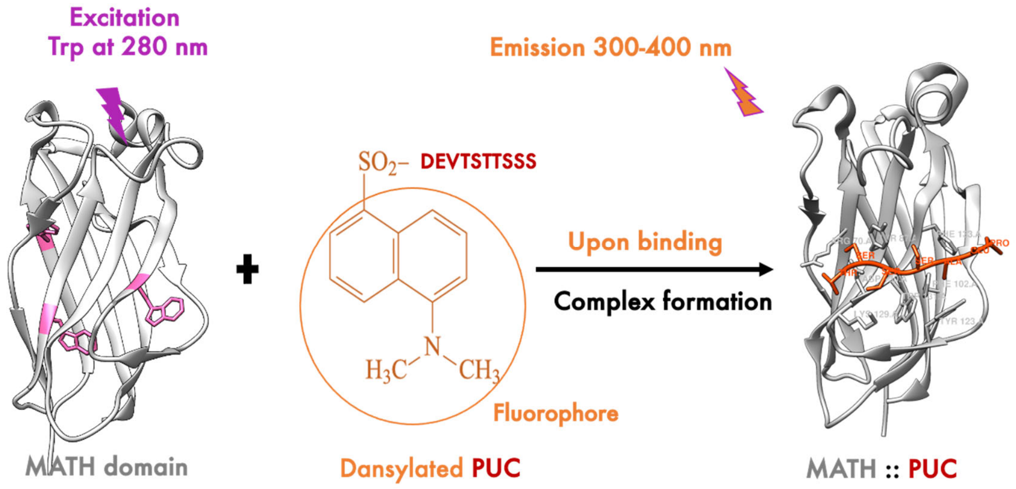

Figure S2. Schematical representation of the FRET signal occurring upon MATH binding to dansylated peptides mimicking SPOP substrates.

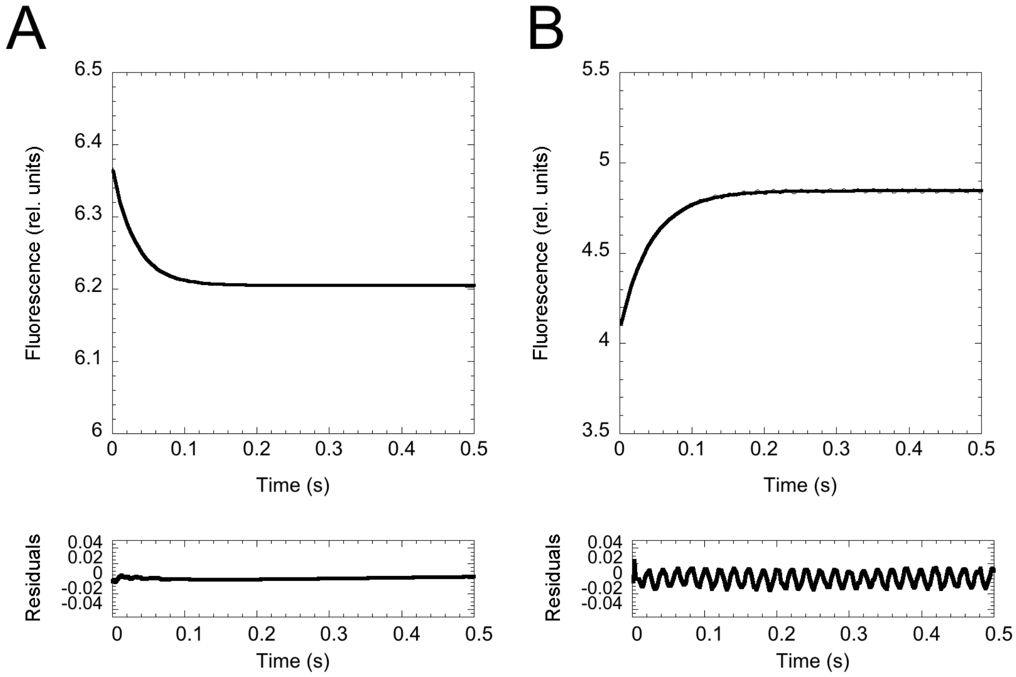

Figure S3. A typical binding (panel A) and displacement (panel B) fluorescence time course observed for the MATH domain when recognizing its substrates. The reported time courses refer to the average of 3-5 independent measurements. The residuals of the fit are indicated below each graph.

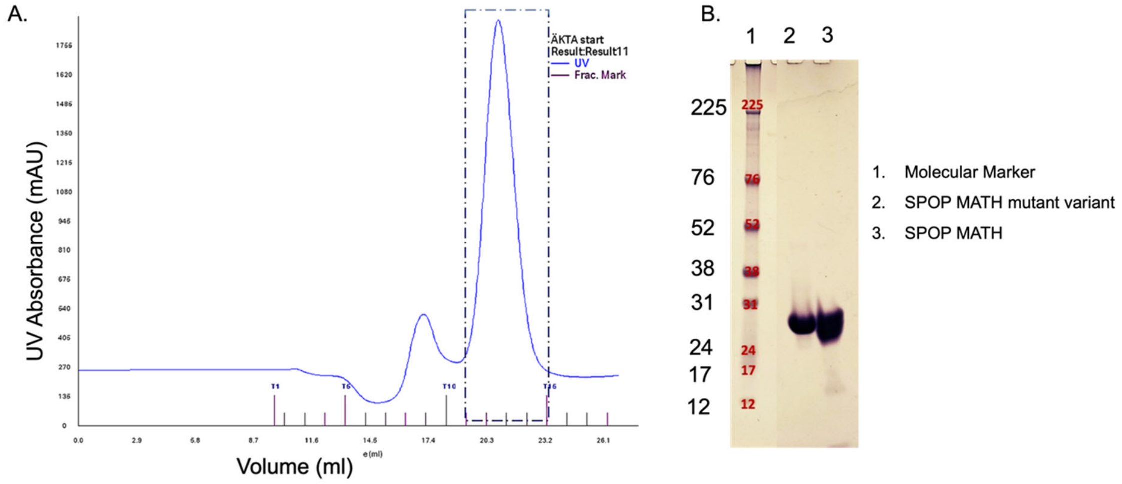

Figure S4. Verifying the purity of SPOP MATH domain variants. The proteins are purified following IMAC His-tag procedure, and the fractions are eluted with an AKTA-prime system. The panel A. represents a typical AKTA MATH domain variant elution profile where the pic highlighted in black rectangle correspond to the elution of a MATH domain variant. The panel B. shows the SDS-PAGE gel of eluted mutant and pseudo wildtype variants of SPOP MATH domain, previously collected, and loaded in prepacked Invitrogen SDS PAGE gel. The Prestained ladder is used as molecular weight marked (1), the mutant and pseudo wildtype variants of SPOP MATH domain are loaded in well 2 and 3, respectively.

Table S1. Equilibrium Unfolding experiements on MATH domain of SPOP variants.

MATH variants	m-value (kcal $mol^{-1}M^{-1}$)	Midpoint (d) (M)	ΔG_{eq} (kcal $mol^{-1)}$	$\Delta\Delta G_{eq}$ (kcal $mol^{-1)}$
WT	1.6 ± 0.6	3.0 ± 0.1	4.8 ± 0.6	-
V30A	1.1 ± 0.1	2.5 ± 0.1	2.75 ± 0.14	-2.05 ± 0.9
V31A	1.7 ± 0.11	2.4 ± 0.04	4.08 ± 0.12	-0.72 ± 0.6
V52A	1.5 ± 0.08	2.8 ± 0.04	4.2 ± 0.09	-0.6 ± 0.6
T57S	1.8 ± 0.1	2.9 ± 0.03	5.22 ± 0.10	0.42 ± 0.61
L66A	1.17 ± 0.04	3.0 ± 0.03	3.51 ± 0.05	-1.29 ± 0.60
V72A	1.7 ± 0.11	2.9 ± 0.03	4.93 ± 0.11	0.13 ± 0.61
L77A	1.7 ± 0.05	3.1 ± 0.01	5.27 ± 0.05	0.47± 0.60
L87A	1.5 ± 0.07	2.7 ± 0.03	4.05 ± 0.08	-0.75 ± 0.60
L90A	1.4 ± 0.2	2.7 ± 0.1	3.78 ± 0.22	-1.02 ± 0.64
I107V	1.6 ± 0.13	2.7 ± 0.05	4.32 ± 0.14	-0.48 ± 0.62
A110G	1.6 ± 0.08	2.7 ± 0.03	4.32 ± 0.09	-0.48 ± 0.61
A117G	1.6 ± 0.07	2.7 ± 0.03	4.32 ± 0.08	-0.48 ± 0.6
V127A	1.8 ± 0.1	2.5 ± 0.02	4.8 ± 0.10	0 ± 0.61
I138V	1.7 ± 0.05	2.9 ± 0.01	4.93 ± 0.05	0.13 ± 0.6
V165A	1.9 ± 0.2	2.9 ± 0.05	5.51 ± 0.21	0.71 ± 0.63
V169A	1.6 ± 0.04	3.0 ± 0.02	4.86 ± 0.04	0.06 ± 0.6
I171V	1.7 ± 0.08	2.9 ± 0.02	4.93 ± 0.08	0.13 ± 0.61

The m-values and midpoint for each experiments, as well as the variations in free energy ($\Delta\Delta G_{eq}$) between the pseudo-wt and each mutant variant are reported in this table. The m-value measures the cooperativity of the folding, whereas the midpoint represents a solution equally populated of native and unfolded proteins.
